# Magnetic nanoparticle-based combination therapy: Synthesis and in vitro proof of concept of CrFe_2_O_4_- rosmarinic acid nanoparticles for anti-inflammatory and antioxidant therapy

**DOI:** 10.1371/journal.pone.0297716

**Published:** 2024-08-06

**Authors:** Afnan Al-Hunaiti, Malek Zihlif, Tuqa Abu Thiab, Wajdy Al-Awaida, Hamzeh J. Al-Ameer, Amer Imraish

**Affiliations:** 1 Department of Chemistry, School of Science, The University of Jordan, Amman, Jordan; 2 Department of Pharmacology, School of Medicine, The University of Jordan, Amman, Jordan; 3 Department of Biological Sciences, School of Science, The University of Jordan, Amman, Jordan; 4 Department of Biology and Biotechnology, American University of Madaba, Madaba, Jordan; 5 Department of Biotechnology, Faculty of Allied Medical Sciences, Al-Ahliyya Amman University, Amman, Jordan; Helwan University, EGYPT

## Abstract

Magnetic drug delivery systems using nanoparticles present a promising opportunity for clinical treatment. This study explored the potential anti-inflammatory properties of RosA- CrFe_2_O_4_ nanoparticles. These nanoparticles were developed through rosmarinic acid (RosA) co-precipitation via a photo-mediated extraction technique. XRD, FTIR, and TEM techniques were employed to characterize the nanoparticles, and the results indicated that they had a cubic spinel ferrite (FCC) structure with an average particle size of 25nm. The anti-inflammatory and antioxidant properties of RosA- CrFe_2_O_4_ nanoparticles were evaluated by using LPS-induced raw 264.7 macrophages and a hydrogen peroxide scavenging assay, respectively. The results showed that RosA- CrFe_2_O_4_ nanoparticles had moderate DPPH scavenging effects with an IC50 value of 59.61±4.52μg/ml. Notably, these nanoparticles effectively suppressed the expression of pro-inflammatory genes (IL-1β, TNF-α, IL-6, and iNOS) in LPS-stimulated cells. Additionally, the anti-inflammatory activity of RosA- CrFe_2_O_4_ nanoparticles was confirmed by reducing the release of secretory pro-inflammatory cytokines (IL-6 and TNF-α) in LPS-stimulated macrophages. This investigation highlights the promising potential of Phyto-mediated CrFe_2_O_4_-RosA as an anti-inflammatory and antioxidant agent in biomedical applications.

## Introduction

Inflammation is the body’s beneficial response to injury or infection. It plays a crucial role in pathology by helping to restore cellular homeostasis and tissue structure and function [1]. The inflammatory response is mediated by two primary components of the host’s defense mechanisms: innate and adaptive immune responses. Innate immunity is the primary response to any foreign material, while adaptive immunity helps eliminate pathogens in the later phase and generates immunological memory. In most cases, inflammation is initiated short time in a host with a functional innate immune system when it encounters foreign stimuli. Acute inflammation is less severe and confined only to a particular area, while chronic inflammation occurs due to the failure to eliminate or destroy the pathogen responsible for causing acute inflammation. However, prolonged inflammation can cause more harm than benefit as it leads to systemic effects mediated by excessive production of cytokines and coagulation factors [2]. This excessive production can induce hepatocytes to facilitate the production of prostaglandins and acute-phase proteins like C-reactive protein. Chronic inflammation can eventually lead to rheumatoid arthritis and, in some cases, cancers as well. Therefore, it’s essential to understand the mechanisms involved in upregulating inflammation, such as NF-κB and COX-2 pathways, to prevent persistent inflammation and its harmful effects [3].

**Fig 1:**
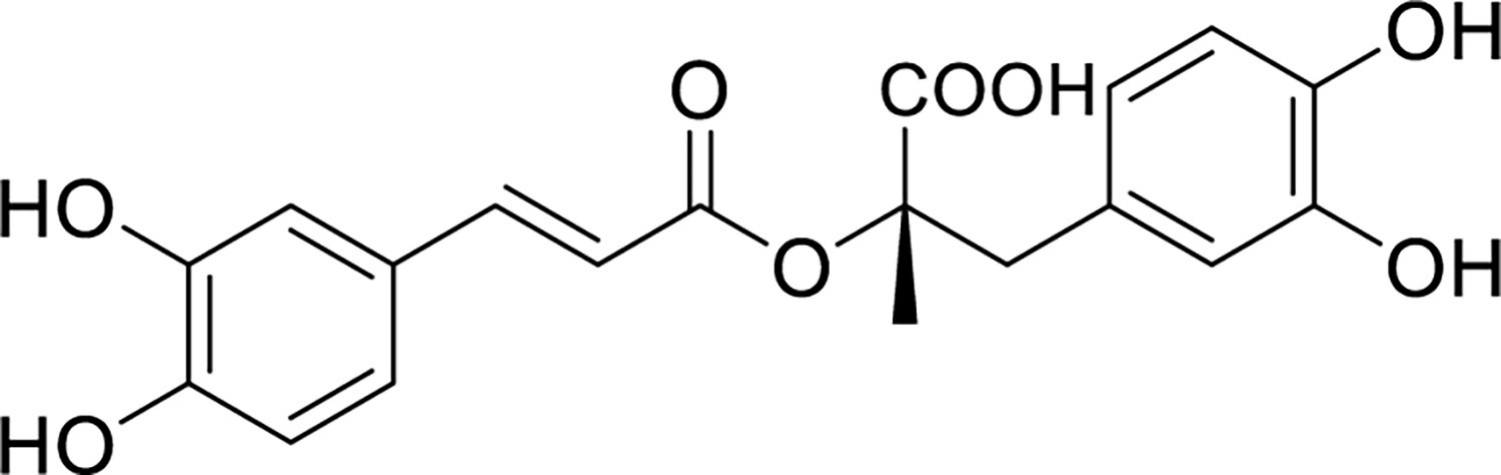
Structure of RosA.

Nanoparticles (NPs) have recently emerged as a potential anti-inflammatory agent. Due to their large surface area to volume ratio, they are better at blocking inflammation-enhancing agents like cytokines and enzymes compared to their bulk counterparts. Various metal and metal oxide NPs have anti-inflammatory properties, including silver, gold, selenium, copper, nickel, zinc oxide, magnesium oxide, iron oxide, and titanium dioxide [4–6]. In this context, magnetic chromium iron oxide nanoparticles (CrFe_2_O_4_ NPs) have drawn attention recently due to their remarkable magnetic and catalytic properties. These nanoparticles exhibit compelling magnetic attributes, rendering them appealing for a broad spectrum of uses, including hyperthermia therapy, magnetic separation, and selective drug delivery. Additionally, the catalytic capabilities of CrFe_2_O_4_ NPs make them valuable in various catalytic reactions [7]. However, their potential cytotoxicity has constrained their utilization in biological applications [1, 8, 9]. Researchers have been exploring surface modification of magnetic nanoparticles using natural compounds to overcome the limitations of traditional methods. Synthesizing nanoparticles using organic sources eliminates the need for toxic chemicals as reducing and capping agents and provides an eco-friendly and cost-effective nanoparticle synthesis method [2, 10]. Plant-mediated ‘green’ synthesis of nanoparticles is one of the most preferred options since it usually requires a neutral pH and occurs at ambient temperatures [11]. Furthermore, plant extracts contain polyphenolic and flavonoids that can reduce and stabilize metal ions into nanoparticles during this process [12]. In a recent study, CrFe_2_O_4_ NPs were synthesized using rosmarinic acid as a vital component of the green synthesis process. Rosmarinic acid is an ester derived from caffeic acid, typically found in plant species belonging to the Boraginaceae family and the Nepetoideae subfamily of the Lamiaceae family. Rosmarinic acid, found in medicinal herbs, plants, and spices, has been associated with various health benefits **Fig 1**. In plants, it serves as a defense compound, while in humans, it exhibits a range of biological activities, including antiviral, antibacterial, antioxidant, antimutagenic, and anti-inflammatory properties. Its anti-inflammatory properties are attributed to its ability to inhibit pro-inflammatory cytokines such as interleukin (IL)-1β, tumor necrosis factor-alpha (TNF-α), and IL-6 [13, 14].

Researchers have been investigating the formulation of Rosmarinic acid (RosA) in the nanoparticles (NPs) as a potential solution for exceptional colloidal stability and minimal cytotoxicity. This could make them highly useful for various biomedical applications. One of the studies conducted by Chen et al. utilized a solvothermal technique to synthesize RosA-coated chromium iron oxide (CrFe_2_O_4_) nanoparticles, which had an average size of around 20nm [15, 16]. These NPs were biocompatible, making them a promising choice for biomedical purposes. In this context, we have synthesized RosA-coated CrFe_2_O_4_ nanoparticles using the co-precipitation method and have tested their potential as anti-inflammatory and anti-oxidant agents. Our findings indicate that the synthesized nanoparticles exhibit superior antioxidant and anti-inflammatory activity and lower cytotoxicity compared to rosmarinic acid or the magnetic CrFe_2_O_4_ particles, establishing them separately as promising contenders for prospective therapeutic applications [17].

## Materials and methods

Iron (III) nitrate (Fe(NO_3_)_3_·9H_2_O), chromium (II) nitrate (Cr(NO_3_)_3_·9H_2_O), rosmarinic acid (RosA), and all solvents employed in the experimental procedures were sourced from Sigma-Aldrich and utilized without further modification. Furthermore, citric acid, sodium hydroxide, and distilled water were incorporated into the practical processes. The crystallographic structure of the MFe_2_O_4_ nanoparticles was determined using (P-XRD) Powder X-ray diffraction, while electron microscopy imaging was employed to assess particle morphology and size distribution. Data acquisition was conducted using a PANalytical X’Pert PRO diffractometer, utilizing monochromatic Cu-Kα radiation (λ = 0.15406 nm) within the angular domine of 20° ≤ 2θ ≤ 100° with 0.02° increments. The NP’s field emission scanning electron microscopy (SEM) images were also acquired using the FEI QUANTA 200 instrument. The FTIR spectra, spanning the 4000–400 cm^−1^ spectral window, were determined with a Perkin-Elmer FT-IR spectrophotometer using KBr disks. Thermal gravimetric analysis (TGA) was performed on synthesized nano ferrite samples, using a 40 mg sample with a heating rate of 10°C /minute rang 20–800°C. The hydrodynamic particle size and size distribution of the nanoparticles (NPs) were assessed using dynamic light scattering (DLS) measurements conducted with the Zetasizer NanoZSP instrument from Malvern Instruments in Worcestershire, UK. The experiments were conducted at a controlled room temperature of 25 ± 2°C. Before analysis, the NP suspensions were appropriately diluted in distilled water at a 1:20 (v/v) ratio and transferred into a cuvette for examination. Size measurements, including the determination of the polydispersity index (PDI) as an indicator of the size distribution of the NPs, and zeta potential measurements, were measured in triplicate to confirm accuracy and reproducibility.

### Synthesis of CrFe_2_O_4_-NPs

A solution was formulated by dissolving Fe(NO3)3⋅9H2O and Cr(NO3)3⋅9H2O at a 2:1 mole ratio in 130 ml of ethanol at room temperature(rt). Subsequently, 2.5 grams of citric acid were introduced into the solution while ensuring continuous stirring. After 30 minutes of vigorous stirring, the suspension’s pH was precisely calibrated to 12.0 by gradually adding an aqueous sodium hydroxide (NaOH) solution. Continuous stirring was maintained for an additional 2-hour period at rt. The solution was then transferred to a 400 ml autoclave and heated at 180°C for 4 hours. Upon completion of this thermal treatment, the resulting precipitates were filtrated, followed by thorough washing with distilled water and ethanol. Subsequently, the collected material was dried at 80°C for 6 hours. The collected powder was calcinated at 1100°C for 4 hours for a pure crystalline product. Finally, the product was examined for its structural and morphological attributes, employing Powder X-ray diffraction (P-XRD) and scanning electron microscopy (SEM) techniques.

### Synthesis of RosA-CrFe_2_O_4_ -NPs

To incorporate Rosmarinic acid (RosA) onto CrFe_2_O_4_-NPs, RosA was used as a reductive agent in the fabrication of spinel iron oxide. In a 1:1 mole ratio, Fe(NO_3_)_3_⋅9H_2_O and Cr(NO_3_)_3_⋅3H_2_O were dissolved in 150 ml of ethanol, stirring at rt. Subsequently, 0.5 grams of RosA were introduced into the ethanolic solution while continuous stirring was maintained. The suspension was gone for 30 minutes, after which NaOH (aqueous) was gradually added until the pH reached 7. Stirring was prolonged for an additional 2 hours at rt. Following this step, the mixture was transferred to a 200 ml autoclave and heated at 120°C for 6 hours. The resulting precipitates were separated via filtration, thoroughly washed using distilled water and ethanol, and heated at 200°C for 6 hours. The collected powder was then subjected to structural and morphological analysis utilizing Powder X-ray diffraction (P-XRD) and scanning electron microscopy (SEM) techniques.

### Cell culture and maintenance

To evaluate the anti-inflammatory effect of RosA- CrFe2O4 NPs, murine macrophage cell line Raw 264.7 (TIB-71™, USA) was used. Raw 264.7 cells were cultivated in Dulbecco’s Modified Essential Medium-DMEM (Gibco) supplemented with 10% fetal bovine serum (Gibco), 1% penicillin-streptomycin (Euroclone), 1% HEPES (Euroclone) and 1% L-Glutamine (Euroclone). The cells were cultured at 37°C with humidity and 5% CO_2_.

### Cytotoxicity of RosA-CrFe_2_O_4_ on Raw macrophages

To test the cytotoxicity of RosA- CrFe_2_O_4_ NPs, a radioactive cell proliferation MTT Assay was performed. 7×10^3^ of Raw cells were seeded in 96-well plates and permitted to adhere overnight. Cells were treated with RosA- CrFe_2_O_4_ NPs and pure RosA extract. All stocks were prepared in DMSO with a 20mg/ml concentration. Seeded cells were treated with a serial dilution of each, starting from a concentration of 200μg/ml, then 100, 50, 25, 12.5, and 6.25μg/ml. The percentage of DMSO was 1% per well. Control wells were present in each plate and treated with 1% DMSO only. Plates were incubated for 72 hours, then IC50 was recorded using CellTiter 96® Non-Radioactive cell proliferation Assay MTT as per manufacturer’s instructions (Promega. USA), and plates were read at 570nm.

### Evaluation of RosA-CrFe_2_O_4_ NPs anti-inflammatory activity against Raw 264.7 murine macrophages

A 2×10^5^ of Raw cells were seeded into 12-well plates and incubated overnight, allowing them to attach. To test the protective anti-inflammatory effect of RosA-CrFe2O4 NPs, three concentrations have been chosen for RosA- CrFe_2_O_4_ NPs and RosA, which are 1/2.5, 1/5, and 1/10 value of IC_50_ for each type. These concentrations were determined based on the cytotoxicity assay, as they will be safe on cells and provide a dose-response curve for the NPs and RosA. For RosA- CrFe_2_O_4_ NPs, concentrations will be as follows: 60, 30, and 15 μg/ml, representing 1/2.5, 1/5, and 1/10 the value of IC_50_, respectively. For pure rosmarinic acid, 12.04, 6.02, and 3.01 μg/ml concentrations will be used as the 1/2.5, 1/5, and 1/10 of the IC_50_ value, respectively. Dexamethasone served as a positive reference for the experiment, with a 40μg/ml concentration. Treatment of cells first started with the different concentrations of RosA-CrFe2O4 NPs and RosA and incubated for 1 hour; then Raw 264.7 cells were induced by 100 ng/ml LPS (Sigma, USA) for 6 hours. Media of each well was obtained and preserved at -80°C for subsequent ELISA study. Cells were harvested for total RNA isolation to proceed with gene expression experiments.

### RNA extraction and evaluation of changes in the expression of pro-inflammatory cytokine genes

Total RNA was extracted using RNeasy Micro Kit (Qiagen, Germany) and measured using Qubit4 (Thermofisher, USA). 500ng of total RNA was converted to cDNA following the manufacturer protocol of (Takara). Briefly, the ready mix was added to 500ng of RNA and incubated for 15 minutes at 37°C, then the reaction was terminated at 85°C for 5 seconds. For the objective of our research, Real-time PCR was performed to examine changes of expression for many pro-inflammatory markers and mediators, including IL-1β, TNF-α, IL-6, and iNOS. To normalize the data the reference gene GAPDH was used for normalization. Table 1 shows a list of primers and their sequences. For a real-time PCR experiment, 11ng of cDNA and 500ng of both forward and reverse primers were mixed with SYPR green master mix (Applied Biosystems, USA). ΔΔCT was calculated for collected data and further analysed using GraphPad Prism statistical analysis software.

**Table 1:** Sequence of forward and reverse primers of different genes.

Gene	Forward primer	Reverse primer
IL-1β	TGCCACCTTTTGACAGTGATG	CCCAGGTCAAAGGTTTGGAAG
TNF-α	GGCCTCCCTCTCATCAGTTC	GGTGGTTTGCTACGACGTG
IL-6	TCTCTGCAAGAGACTTCCATCC	TGAAGTCTCCTCTCCGGACTT
iNOS	AAGGGTCCCTGAGGGCTGTA	GGGCTTTGCTTCACTAGAGCG
GAPDH	GGGTCCCAGCTTAGGTTCAT	TACGGCCAAATCCGTTCACA

### Determination of cytokine release

Raw 264.7 cells were stimulated using LPS derived from E. coli 0111:B4 (Sigma, USA). Raw 264.7 cells were initially stimulated with 100ng/mL of LPS for 6 hours, utilizing 12-well plates for the cell culture. Later, the cells were exposed to Rosmarinic acid-coated chromium iron oxide nanoparticles (RosA- CrFe_2_O_4_ NPs) for 1 hour. Subsequently, the cells were further incubated with LPS at 37°C for 6 hours. A control group, referred to as the LPS control, was solely treated with LPS. Following the 6-hour incubation period, the supernatants from the cell cultures were carefully extracted and transferred to clean tubes. These samples were subsequently stored at -80°C pending the subsequent assessment of cytokine secretion. The quantification of pro-inflammatory cytokines, specifically IL-1β and TNF-α, was conducted utilizing enzyme-linked immunosorbent assay (ELISA) kits, following the procedures outlined by the manufacturer.

### DPPH radical scavenging activity assay

The DPPH radical scavenging assay was conducted following the protocols established by Sharififar et al. [18]. Specifically, 50μl of varying concentrations of the nanoparticles (NPs) were combined with 5ml of 0.004% 2,2-Diphenyl-1-picrylhydrazyl (DPPH) dissolved in methanol. This mixture was subsequently incubated in a dark environment at room temperature. After a 30-minute incubation period, the absorbance was recorded at 517nm against a methanol reference. The same procedure was employed using ascorbic acid as a standard.

### Nitric oxide scavenging activity assay

The Griess assay was utilized to quantify nitric oxide (NO) liberation from sodium nitroprusside. Sodium nitroprusside is recognized for its degradation in a physiologically relevant aqueous solution with a pH of 7.2, yielding NO. In aerobic conditions, NO reacts with oxygen, forming stable end products, namely nitrate and nitrite, which are subject to quantification via the Griess reagent. To initiate the process, sodium nitroprusside (5mM) was gently mixed with various concentrations of extracts (dissolved in dimethyl sulfoxide) in phosphate-buffered saline and allowed to incubate for 2.5 hours at room temperature (RT). After incubation, 500μl portions from each sample were combined with an equal volume of the Griess reagent. Subsequently, the absorbance of the reaction mixture was measured at 546 nm, and this measurement was normalized by referencing it to the absorbance of potassium nitrite, which had undergone identical treatment with the Griess reagent (2–3).

### ABTS radical scavenging assay

The protocol for assessing the scavenging ability against the free radical cation (ABTS+), utilizing 2,2ʹ-casino-bis (3-ethylbenzothiazoline-6-sulphonic acid), was conducted following the established methodology [19, 20]. An aqueous ABTS solution (7 mM) was combined with an aqueous potassium persulfate solution (2.45 mM) in equal proportions to prepare the stock solution. This combination was permitted to react at ambient temperature without light for 12–16 hours. Various concentrations of nanoparticles dissolved in absolute alcohol were subsequently prepared. These nanoparticle samples were introduced into the ABTS solution and incubated for 30 minutes. The absorbance of the resulting mixture was quantified at a wavelength of 734nm. It’s worth noting that the same procedure was replicated using ascorbic acid as a standard reference (4–5).

### Statistical analysis

Statistical analysis of the data was performed using GraphPad Prism 8 software. The results were expressed as mean ± standard error of the mean (SEM). The significance of the anti-inflammatory effects of RosA- CrFe_2_O_4_ NPs was measured using the student’s t-test. Values were statistically significant at p < 0.05.

## Results and discussion

### Characterization of bimetallic nanoparticles

**Fig 2:**
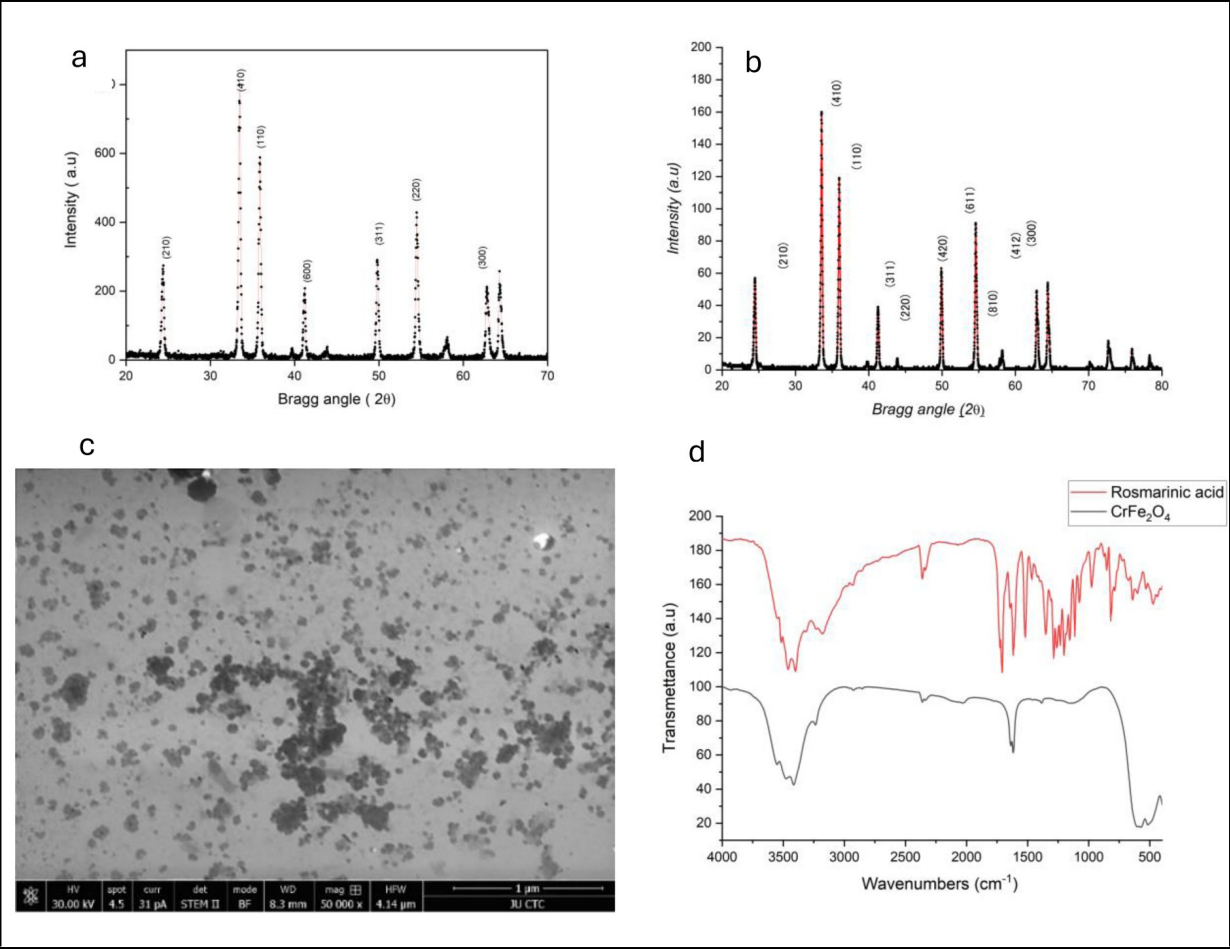
XRD pattern of (a) CrFe_2_O_4_ (b) RosA- CrFe_2_O_4_ NPs. (c) SEM image of RosA- CrFe_2_O_4_ NPs, FT-IR spectra of NPs (RosA and RosA- CrFe_2_O_4_).

Fig 2(A) shows the XRD pattern obtained from CrFe_2_O_4_-NPs, validated by standard JCPDS card no (00–002–1357). The cubic spinel ferrite structure (MeFe_2_O_4_, where “Me” represents a divalent ion) consists of metal ions, with one ion per molecule occupying the tetrahedral (A) site and two ions per molecule occupying the octahedral (B) site. The X-ray diffraction (XRD) patterns of CrFe_2_O_4_ (JCPDS file No: 00–002–1357) reveal reflections at 18°, 28°, 34°, 36°, 42°, 47°, 54°, 59°, 63°, and 68, which reflects (210), (410), (110), (200), (311), (220), (300) indicating the presence of a spinel cubic structure (fcc). The intense and sharp peaks suggest that the products were well-crystallized [21]. The diffraction peaks exhibit a high signal-to-noise ratio, and the phases are identified by their corresponding Miller indices (hkl). Bragg’s law relates the d-spacing between a set of parallel atomic planes in the crystal lattice to a diffraction peak at angular position (2θ) Eq 1.


2dsinθ=λ (1)

These peaks indicated the presence of a cubic spinel structure with a lattice constant a of a = 0.8 Å with average particle size (24.35± 0.007) Å, while after incorporating RosA, the pattern showed the same with particle size (29.67± 0.008).

The morphology of RosA-CrFe_2_O_4_ nanoparticles was examined using scanning electron microscopy (SEM), and a cubic structure with a particle size of 25nm was observed (Fig 2C). Further characterization was carried out using FT-IR spectroscopy (Fig 2D). The FTIR spectra of RosA and RosA-CrFe_2_O_4_ are presented in Fig 2D. The RosA spectra displayed peaks at 3420 cm-1, corresponding to the O-H groups in the carboxylic acid of RosA. The bands observed at 2923 cm^-1^ and 2855 cm^-1^ were assigned to the stretching vibrations of C-H groups in the RosA. On the other hand, the FTIR of RosA-CrFe_2_O_4_ NPs revealed peaks at 3420 cm^-1^, corresponding to the stretching vibration of O-H groups in the carboxylic acid of RosA. The spectral features seen at 2923 cm^-1^ were attributed to the asymmetric and symmetric stretching vibrations of C-H groups in the RosA, respectively, thus confirming the presence of RosA in CrFe_2_O_4_ NPs. Additionally, the FTIR displayed a characteristic peak at 580 cm^-1^ and 640 cm^-1^, indicating the Fe-O bond stretching vibration in the spinel structure of CrFe_2_O_4_. These peaks were also observed in the spectra of the RosA-CrFe_2_O_4_ NPs, which suggested that the spinel structure was preserved after coating with RosA [21, 22]. All the spectra provide good evidence of the incorporation of the RosA in CrFe_2_O_4_ nanoparticles.

### DLS and zeta potential of the synthesized nanoparticles

The particle size, PDI, and zeta potential of the prepared NPs were determined by analyzing the electrophoretic mobility of the nanoparticle dispersions. The outcomes are presented in Table 2, indicating that all samples exhibited a PDI of less than 0.5, suggesting a relatively narrow size distribution of particles. The zeta potential values for Sample 1, 2, and 3 were determined to be -40.35, -21.42, and -35.6. These findings indicate that the nanoparticles possess strong anionic surface properties, as zeta potentials below -30 mV are typically classified as strongly anionic [23]. Due to aggregation, RosA particles have a large size. However, particle size decreases when combined with metallic NP.

**Table 2:** Zeta potential, signal-to-noise ratio, and sizes of nanoparticles determined by analyzing the electrophoretic mobility of the nanoparticle dispersions.

Entry	Sample	Zeta potential (mv)	PDI	Signal/Noise Ratio	Z-Average (nm)
1	CrFe_2_O_4_	-40.35	0.134	0.463±0.045	24. 3
2	Rosa	-21.42	0.268±0.058	0.493±0.004	105.7
3	RosA-CrFe_2_O_4_	-35.60	0.186±0.146	0.231±0.033	37.55

### Cell viability of Raw 264.7 cells treated with RosA-CrFe_2_O_4_ NPs

Cell viability assay is essential to determine the cytotoxic impacts of synthesized nanoparticles (NPs) on various cell lines *in vitro*. In the present study, an MTT assay was employed to evaluate the cytotoxicity of RosA- CrFe_2_O_4_ NPs on the Raw 264.7 macrophages. Macroscopic cell viability remained unaffected by the RosA- CrFe_2_O_4_ NPs at concentrations lower than 100μg/ml. The percentage viability of Raw 264.7 cells was 75%, 82%, and 85% when the cells were subjected to 60, 30, and 15μg/ml of RosA- CrFe_2_O_4_ NPs, respectively. Therefore, 60, 30, and 15μg/ml of RosA- CrFe_2_O_4_ NPs were the least toxic concentrations to examine their anti-inflammatory activity. Results have shown that by increasing the concentration of NPs, the percentage of inhibition of cell growth increased, while RosA- CrFe_2_O_4_-NPs did not induce a significant reduction in the viability of macrophage cells. These data are consistent with a study demonstrating the cytotoxicity of CrFe_2_O_4_-NPs at high concentrations (250 μg/ml) against macrophage cells [24]. Another similar study investigated the effect of CrFe_2_O_4_-NPs on human macrophages. Results have shown no toxic activity with an IC50 > 416.5 μg/ml [25]. Our findings suggest that phyto-synthesis of CrFe_2_O_4_ NPs could offer protection against cell toxicity by masking the metallic NPs’ lower cytotoxic effect on normal cells by replacing the hazardous chemicals used in conventional methods with naturally occurring reducing agents of plant-derived material.

### Inhibition of LPS-induced inflammatory response by RosA- CrFe_2_O_4_ NPs on Raw264.7 macrophages

Sustainable production of nanoparticles through natural compound-mediated synthesis such as RosA shows various advantages; they play a critical role in furnishing stability and biocompatibility to NPs. LPS constitutes a gram-negative bacteria strain and prompts macrophage participation in the immune response. Several studies have shown that LPS induces the Raw 264.7 cells, and these macrophages were employed as *in vitro* simulations of inflammation to evaluate potential anti-inflammatory agents. To observe the suppressive impact of RosA- CrFe2O4 NPs for treating inflammation, the LPS-induced Raw 264.7 cell line was employed as an *in vitro* model. MTT assay is used to determine the effects of RosA- CrFe_2_O_4_ NPs on the cell viability of Raw 264.7 cells. As previously stated, RosA- CrFe_2_O_4_ nanoparticles (NPs) exhibited no discernible cytotoxicity within the 60 to 15 μg/ml concentration range. Furthermore, RosA- CrFe_2_O_4_ NPs demonstrated a lack of cytotoxic impact at concentrations as high as 100 μg/ml on the tested Raw 264.7 cells. Consequently, subsequent investigations were conducted utilizing 60, 30, and 15 μg/ml concentrations. Numerous studies have documented that suppressing iNOS, IL-1β, IL-6, and TNF-α expressions attenuates the pathogenesis of various disorders, including arthritis and cancer [13]. Additionally, a body of research has elucidated the roles of these cytokines in processes associated with inflammation, tumor formation, and some auto-immune conditions [26].

Because of their pivotal role in the onset of inflammation, downregulation of the expression of iNOS, IL-1β, IL-6, and TNF-α is considered a promising therapeutic approach for effectively treating and preventing inflammatory ailments.

**Fig 3:**
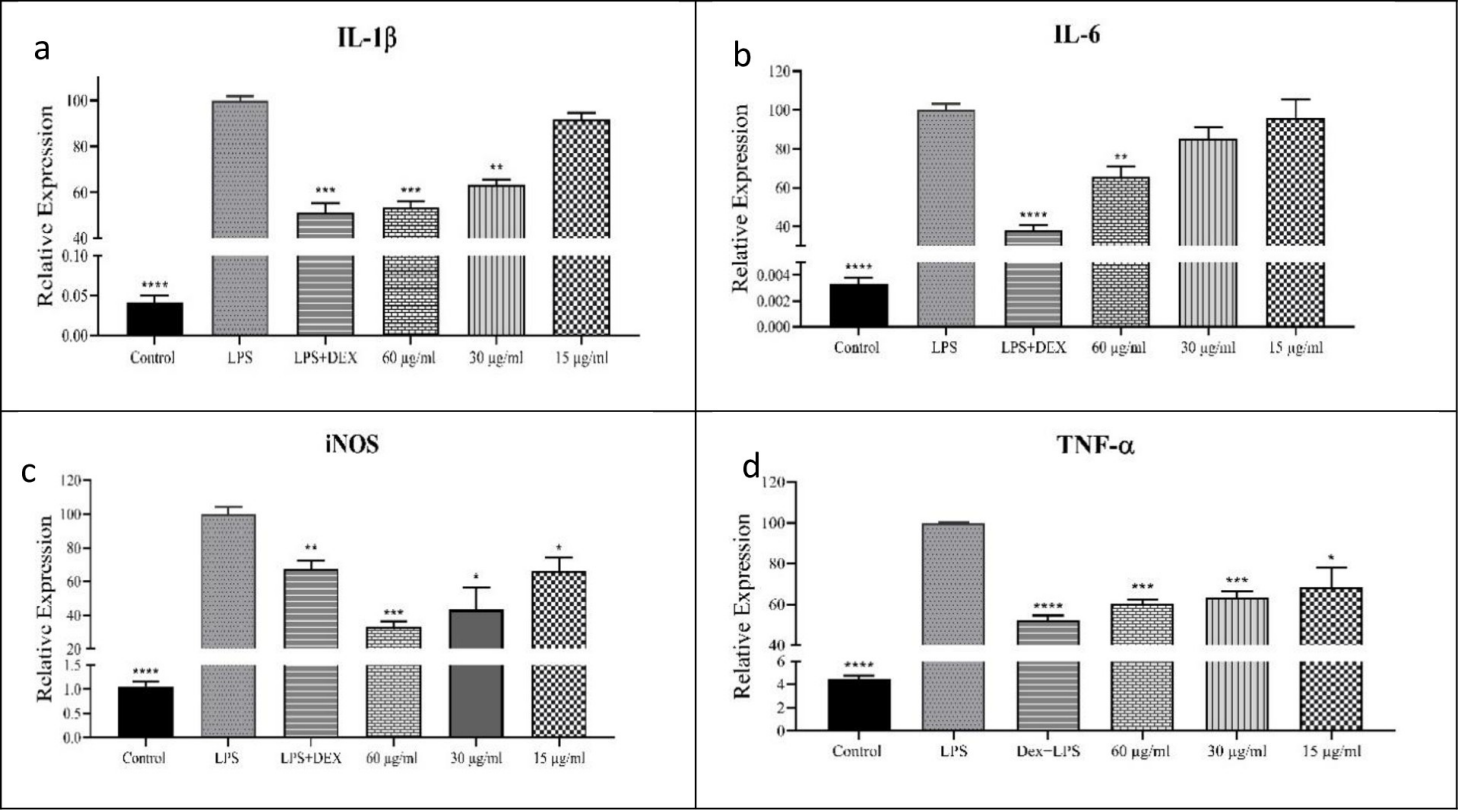
Effects of RosA- CrFe_2_O_4_ NPs on a) IL-1β, b) IL-6, c) iNOS, and TNF-α gene expression. RAW264.7 cells were pre-treated with the defined concentrations of RosA- CrFe_2_O_4_ NPs for one h, then stimulated with LPS (100 ng/ml) for 6 hrs. Accordingly, total RNAs were extracted, and the mRNA expression levels of IL-1β, IL-6, iNOS, and TNF-α were measured by RT-PCR analysis. The data outlined demonstrate mean values of three independent experiments ± SEM. the statistical significance of the RosA- CrFe_2_O_4_ NPs-treated cells to the LPS controls is indicated with * for P<0.05, **for P < .01 and ***for P<0.001.

To examine the inhibitory activity of RosA- CrFe_2_O_4_ NPs on inflammatory mediators, real-time PCR was performed to determine the expression of iNOS, IL-1β, IL-6, and TNF-α in the LPS-induced Raw 264.7 cells before and after treatment with RosA- CrFe_2_O_4_ NPs. Gene expression analysis exhibited that the treatment of high concentration (60μg/ml) of RosA- CrFe_2_O_4_ NPs can inhibit the expression of IL-1β mRNA considerably compared with the LPS-induced control group (Fig 3). RosA- CrFe_2_O_4_ NPs showed approximately 36.4% and 8% inhibition of IL-1β mRNA levels at 30μg/ml and 15μg/ml concentrations of nanoparticles, about (Fig 3A). In addition, our findings demonstrate that RosA- CrFe_2_O_4_ NPs significantly minimized the mRNA levels of LPS-mediated inflammatory gene IL-6 in RAW264.7 cells at 60μg/ml (Fig 3B). Interestingly, our synthesized NPs unassumingly impacted the outflow of IL-6 contrasted with other tested pro-inflammatory cytokines. This can be expected by the contribution of this cytokine in numerous immunological processes [27]. Along with blending with different cytokines like TNF-α and IL-1β, it plays a key modulator of immunological reactions set off by exogenous microorganisms, including bacterial and viral poisons. Moreover, IL-6 cytokine is an immediate objective for the transcription factor nuclear factor-kappa B (NF-κB), an endless supply of immunological pathways, synergistic cooperation between the signal transducer and activator of record 3 (STAT3) and NF-κB causes an upregulation of NF-κB itself, which thusly enhances IL-6 expression levels. This positive criticism circle among STAT3 and NF-κB is known as the IL-6 enhancer, permitting IL-6 to assume a part in regulating other immunological reactions [28].

The lowest concentration used to evaluate the anti-inflammatory effect of RosA- CrFe_2_O_4_ NPs decreased the mRNA levels of iNOS and TNF- α significantly (Fig 3C and 3D). Therefore, it suggested that RosA- CrFe_2_O_4_ NPs have anti-inflammatory actions by suppressing the expression of pro-inflammatory genes in Raw264.7 cells stimulated by LPS.

Rosa- CrFe2O4 NPs suppress the production of pro-inflammatory cytokines like IL-1β and TNF-α and also cause a reduction in iNOS and IL-6 gene expressions at different concentrations, which assist in achieving its anti-inflammatory potential. To the best of our knowledge, and based on the available literature data, this work represents the first study of the anti-inflammatory potential of green synthesized CrFe2O4 NPs using rosmarinic acid.

These results demonstrate the strong anti-inflammatory properties of our green-synthesized CrFe2O4 NPs and their concentration-dependent inhibition of pro-inflammatory cytokine production. The study’s unique findings are the first to investigate how green synthetic CrFe2O4 NPs affect inflammatory reactions. The results of this investigation, however, highlight the significance of learning how CrFe2O4 NPs work to suppress the expression of pro-inflammatory genes like IL-1β, TNF-α, IL-6, and iNOS. Beyond the fact that they are capped with natural plant-reducing agents (rosmarinic acid) that enable them to perform their synergistic effects, the remarkable safety and anti-inflammatory effectiveness may be linked to the synergistic effect of both chromium and iron.

Furthermore, In the current work, a unique delivery system comprising two distinct metals chromium and ferrite was developed to achieve the anti-inflammatory therapeutic target. To address the cytotoxicity concern, we green synthesized our bimetallic CrFe2O4 NPs using RosA as a natural raw material. Numerous researchers have examined RosA’s anti-inflammatory capabilities. Jiang and his colleagues found that RosA inhibited NF-κB, which in turn prevented the release of pro-inflammatory mediators like TNF-α and IL-6 in LPS-stimulated Raw cells [27]. In addition, when LPS was used to stimulate human umbilical vein endothelial cells (HUVECs), RosA was found to significantly and dose-dependently decrease pro-inflammatory mediators as well as nitric oxide synthase, confirming its anti-inflammatory and antioxidant properties [29]. In this investigation, the combination of RosA and bimetallic NPs demonstrated a biosafe and highly effective anti-inflammatory therapeutic target.

### Cytokines secretion

**Fig 4:**
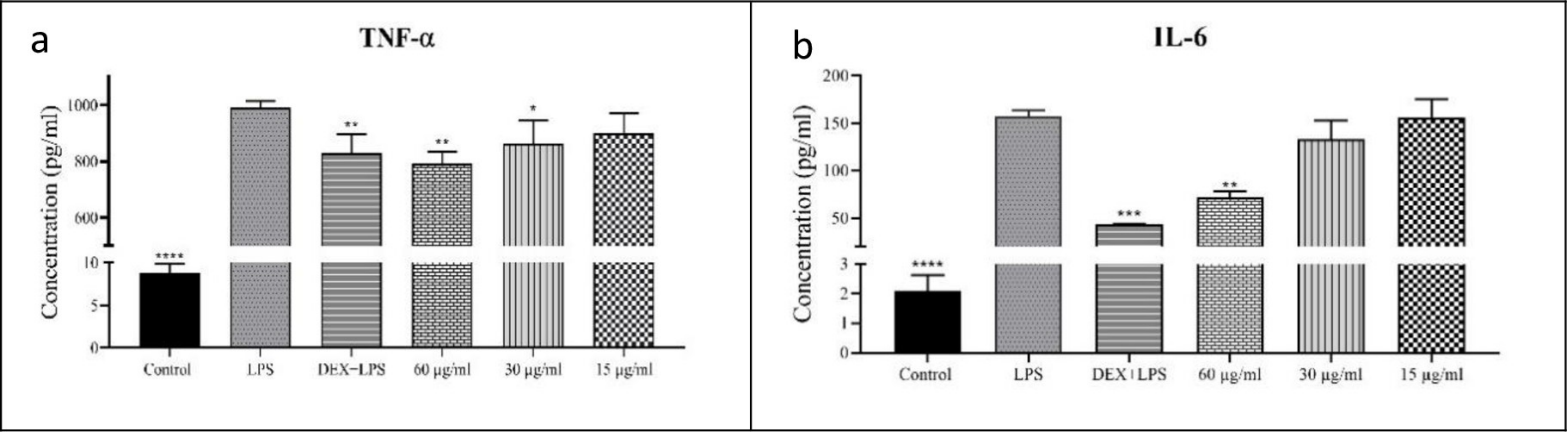
Effects of RosA- CrFe_2_O_4_ NPs on cytokine secretion in LPS-stimulated Raw 264.7 cells. (a) represents TNF-α, and (b) represents IL-6. Each value represents mean ± SEM; the statistical significance of the RosA- CrFe_2_O_4_ NPs-treated cells to the LPS controls is indicated with * for P<0.05, **for P < .01 and ***for P<0.001.

ELISA was used to assess the impacts of RosA- CrFe_2_O_4_ NPs on cytokine production in the Raw 264.7 cells. The macrophages were stimulated with LPS before exposure to 60, 30, and 15μg/mL RosA- CrFe_2_O_4_ NPs. Exposure of Raw 264.7 cells to LPS induction initiated the synthesis of pro-inflammatory cytokines, which play a pivotal role in orchestrating an immune response when generated in appropriate quantities. The graphical representation of cytokine production by the Raw cells in the presence of RosA- CrFe_2_O_4_ NPs is presented in Fig 4. Stimulation of the Raw cells with LPS alone resulted in the robust generation of pro-inflammatory cytokines (TNF-α and IL-6). However, including RosA- CrFe_2_O_4_ NPs (60, 30 and 15 μg/mL) in the LPS-stimulated cells led to a notable reduction in cytokine production levels. Notably, the levels of TNF-α production in the Raw cells treated with 60 and 30μg/ml RosA- CrFe_2_O_4_ NPs were significantly decreased when compared to the LPS-treated cells (Fig 4A).

Furthermore, the concentrations of IL-6 production in the macrophages exposed to 60, 30, and 15 mg/ml RosA- CrFe_2_O_4_ NPs exhibited a significant reduction compared to LPS-induced cells (Fig 4B). Hence, the findings obtained in this study demonstrate the anti-inflammatory properties of RosA- CrFe_2_O_4_ NPs, as evidenced by the diminished secretion of TNF-α and IL-6 cytokines in LPS-activated Raw 264.7 macrophages. CrFe_2_O_4_ NPs synthesized using Boswellia cartei Resin extract have yielded analogous outcomes [10], as they effectively attenuated the levels of pro-inflammatory cytokines (IL-6 and TNF-α) in Raw 264.7 cells, signifying their robust anti-inflammatory potential. These findings validate the impact of CrFe2O4 NPs on mRNA levels and highlight their efficacy as a dose-dependent anti-inflammatory agent on macrophages that have been examined.

Overall, TNF-α, IL-1β, iNOS and IL-6 modulate inflammatory responses and their levels are markedly increased in both acute and chronic inflammation. However, several inorganic metals are essential trace elements for human bodies, where chromium is essential in some physiological roles in human bodies [30] and plays an important role in modulating the immune system. Recently, nanoparticles have been proposed as a potential anti-inflammatory agent due to their biocompatibility and safety [31]. In addition, these small particles are easily uptake by the cell plasma membrane by phagocytosis; allowing them to act as inhibitors of pro-inflammatory agents such as the cytokine pathway in immune cells such as macrophages [32, 33].

### Antioxidant activity of RosA-CrFe2O4 NPs

The IC_50_ value of RosA- CrFe_2_O_4_ NPs on NO scavenging activity was 82.61±6.89 μg/ml. Ascorbic acid served as the benchmark compound, and 21.8 ± 1.9 μg/ml of ascorbic acid was requisite to achieve a 50% inhibitory effect.

The IC_50_ value of RosA- CrFe_2_O_4_ NPs was higher than a reference compound. Also, the IC_50_ value of RosA- CrFe_2_O_4_ NPs on DPPH scavenging activity was 59.61±4.52 μg/ml. compared to standard ascorbic acid (IC_50_ = 5.23±0.052 μg/ml).

## Conclusion

RosA- CrFe_2_O_4_ NPs were successfully synthesized using RosA as a reducing agent using the co-precipitation technique. The formulated RosA- CrFe_2_O_4_ NPs were characterized using XRD, IR, and SEM and demonstrated robust stability in biological environments. These nanoparticles were developed through rosmarinic acid (RosA) co-precipitation via a photo-mediated extraction technique. XRD, FTIR, and TEM techniques were employed to characterize the nanoparticles, and the results indicated that they had a cubic spinel ferrite (FCC) structure with an average particle size of 25nm. The anti-inflammatory and antioxidant properties of RosA- CrFe_2_O_4_ nanoparticles were evaluated by using LPS-induced raw 264.7 macrophages and a hydrogen peroxide scavenging assay, respectively. The results showed that RosA- CrFe_2_O_4_ nanoparticles had moderate DPPH scavenging effects with an IC50 value of 59.61±4.52μg/ml. Notably, these nanoparticles effectively suppressed the expression of pro-inflammatory genes (IL-1β, TNF-α, IL-6, and iNOS) in LPS-stimulated cells. Additionally, the anti-inflammatory activity of RosA- CrFe_2_O_4_ nanoparticles was confirmed by reducing the release of secretory pro-inflammatory cytokines (IL-6 and TNF-α) in LPS-stimulated macrophages. These findings validate the impact of CrFe_2_O_4_ NPs on mRNA levels and highlight their efficacy as a dose-dependent anti-inflammatory agent on macrophages that have been examined. Therefore, our results demonstrate the therapeutic potential of CrFe_2_O_4_-RosA NPs as anti-inflammatory and antioxidant agents. Further studies are ongoing to understand the molecular mechanism of the prepared NPs.
